# Novel Processes for the Extraction of Phenolic Compounds from Olive Pomace and Their Protection by Encapsulation

**DOI:** 10.3390/molecules26061781

**Published:** 2021-03-22

**Authors:** Sofia Chanioti, Maria Katsouli, Constantina Tzia

**Affiliations:** Laboratory of Food Chemistry and Technology, School of Chemical Engineering, National Technical University of Athens, 15780 Zografou, Greece; schanioti@gmail.com (S.C.); mkatsouli@chemeng.ntua.gr (M.K.)

**Keywords:** phenolic compounds, olive pomace, extraction, microwaves, ultrasound, homogenation, high hydrostatic pressure, deep eutectic solvents, microencapsulation, nanoemulsion

## Abstract

Olive pomace, the solid by-product derived from olive oil production consists of a high concentration of bioactive compounds with antioxidant activity, such as phenolic compounds, and their recovery by applying innovative techniques is a great opportunity and challenge for the olive oil industry. This study aimed to point out a new approach for the integrated valorization of olive pomace by extracting the phenolic compounds and protecting them by encapsulation or incorporation in nanoemulsions. Innovative assisted extraction methods were evaluated such as microwave (MAE), homogenization (HAE), ultrasound (UAE), and high hydrostatic pressure (HHPAE) using various solvent systems including ethanol, methanol, and natural deep eutectic solvents (NADESs). The best extraction efficiency of phenolic compounds was achieved by using NADES as extraction solvent and in particular the mixture choline chloride-caffeic acid (CCA) and choline chloride-lactic acid (CLA); by HAE at 60 °C/12,000 rpm and UAE at 60 °C, the total phenolic content (TPC) of extracts was 34.08 mg gallic acid (GA)/g dw and 20.14 mg GA/g dw for CCA, and by MAE at 60 °C and HHPAE at 600 MPa/10 min, the TPC was 29.57 mg GA/g dw and 25.96 mg GA/g dw for CLA. HAE proved to be the best method for the extraction of phenolic compounds from olive pomace. Microencapsulation and nanoemulsion formulations were also reviewed for the protection of the phenolic compounds extracted from olive pomace. Both encapsulation techniques exhibited satisfactory results in terms of encapsulation stability. Thus, they can be proposed as an excellent technique to incorporate phenolic compounds into food products in order to enhance both their antioxidative stability and nutritional value.

## 1. Introduction

Natural antioxidants are bioactive compounds derived from plant sources including vegetables, fruits, grains, herbs, spices and oilseeds [1]. The primary mechanism of action of antioxidant compounds is based on the prevention of an oxidative chain providing radical stabilization and decrease of the oxidative damage in the human body. The antioxidant compounds may either deactivate metals or inhibit the hydroperoxides of lipids contributing to the obstruction of undesirable volatiles generation, as well as to the removal of singlet oxygen [2]. Thus, the antioxidant compounds can be characterized as “those substances that prevent or considerably retard the oxidation of susceptible chemical compounds including fats”.

Natural antioxidant compounds are mainly classified into phenolic compounds, carotenoids, and certain vitamins. Phenolic compounds include simple molecules (gallic acid, caffeic acid, vanillin, etc.) as well as polyphenols (flavonoids) [3]. It has been proved that most phenolic compounds have antimicrobial, antifungal, and anticarcinogenic activity [4]. The main carotenoids are α-carotene, β-carotene, lycopene, and lutein, which show antioxidant activity [5]. Concerning the vitamins with antioxidant activity, the most important are vitamin C, which is present in fruits and vegetables, and vitamin E, a fat-soluble vitamin that is found in legumes and cereal grains [1].

Natural antioxidants have limited uses, mainly, because they present reduced antioxidant efficiency; thus, they are required at high concentration levels, they have undesirable odor and flavor, and present excessive loss during the processing procedure. Due to the above disadvantages, they are occasionally replaced by synthetic chemical compounds, which possess higher antioxidant activity, stability, and availability [6]. Butylated hydroxyanisole (BHA), butylated hydroxytoluene (BHT), and propyl gallate (PG) belong to the category of synthetic phenolic compounds which effectively inhibit the oxidation process. Ethylenediaminetetraacetic acid (EDTA) is a chelating antioxidant agent which reduces the contribution of metals to oxidation by binding them. However, the concern of consumers about the safety of synthetic additives in food products and the need for environmentally friendly and sustainable practices force the food industry to develop sustainable processes for the recovery of natural antioxidant compounds from food sources and by-products [7].

The recovery of antioxidant compounds from different plant sources can be achieved by extraction processes. The quality of the obtained extracts in terms of the type of compounds and their antioxidant efficiency depend on the characteristics of the plant source, including the geographical origin, as well as the handling and the storage conditions, but also on the involved extraction technologies.

**Figure 1:**
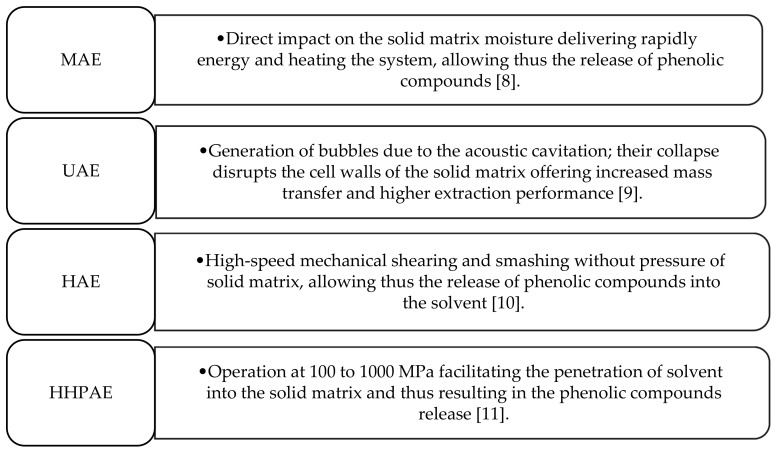
The main mechanism of action of assisted extraction techniques. MAE: microwave- assisted extraction; UAE: ultrasound- assisted extraction; HAE: homogenate- assisted extraction; HHPAE: high hydrostatic pressure- assisted extraction.

Nowadays, there is a trend toward using new strategies for extraction processes for the recovery of antioxidant compounds by introducing microwave-(MAE), ultrasound-(UAE), homogenate-(HAE) and high hydrostatic pressure-(HHPAE) assisted extraction techniques [4]. Innovative assisted extraction techniques enable high extraction performance, while requiring reduced extraction temperature, time, and energy consumption [8,9,10,11,12]. The main mechanism of action of assisted extraction techniques is presented in Figure 1. Moreover, a new generation of solvents has recently been proposed for the extraction of antioxidant compounds from plant sources, called natural deep eutectic solvent (NADES), by combining certain natural components.

Since antioxidant compounds are susceptible to degradation and highly sensitive to various environmental conditions (pH, temperature, oxygen light, and moisture) resulting in losses of their nutritional and functional properties during storage, encapsulation techniques were developed including their incorporation into a delivery system before their introduction into the food matrix [13,14]. Various delivery systems can be designed to have numerous benefits to the food industry: (i) incorporation of active compounds into the food matrix without altering the food quality attributes (appearance, texture, flavor, etc.); (ii) protection of active compounds from chemical, physical, or biological degradation; (iii) masking any bitter or astringent taste; (iv) improving stability of active compounds during transport and storage; (v) improving their ease of handling; and (vi) improvement of product shelf-life. There are different techniques for encapsulation of natural antioxidants that include phase separation, spray drying, freeze-drying, nanoemulsions, liposomal entrapment, coacervation, inclusion complexation, ionic gelation, solvent evaporation, and supercritical fluid precipitation.

Nowadays, the nanoemulsion-based delivery system is one of the most prominent encapsulation techniques providing a wide array of advantages in encapsulating natural antioxidant in food products, such as enhancing chemical stability, and increasing either bioavailability, fortification, or both. During nano-emulsification, two immiscible liquids (usually water and oil) and an emulsifier are converted into a monophase system by using high energy input. Therefore, by using a colloidal system such as nanoemulsion, it is possible to encapsulate various lipophilic and hydrophilic components into different food matrices. Thus, recently many researchers have focused on exploring the nanoemulsions for encapsulation of natural antioxidants [15,16,17,18]. Nanoemulsions are more stable against gravitational separation and aggregation, compared to conventional emulsions, due to their smaller droplet size (<500 nm) and higher liquid droplet interface area. They can also be transparent and exhibit a variety of rheological properties that allow them to modify or design the texture of food products. Various colloidal delivery systems based on emulsification with different structures and properties can be fabricated using different ingredients [19] that may find wide applications in the food and nutrition, biology, and pharmacology areas, especially in the high-efficiency encapsulation and targeted delivery of bioactive ingredients.

This study aimed to present the feasibility of innovative approaches by using assisted extraction methods by microwaves, homogenization, ultrasounds, and high hydrostatic pressure and various solvent systems including ethanol, methanol, and NADESs for the recovery of phenolic compounds from olive pomace. The obtained extracts were evaluated in terms of their total phenolic content (TPC) and antioxidant radical scavenging (DPPH), as well as of their composition in individual compounds by high-performance liquid chromatography (HPLC). Furthermore, through an integrated process, the encapsulation of the phenolic compounds in matrices of maltodextrin by freeze- or spray- drying techniques and in nanoemulsion systems was evaluated in terms of encapsulation stability. The proposed approach could provide an alternative tool for green extraction of phenolic compounds from olive pomace and for their encapsulation in order to develop food products with high antioxidative stability and nutritional value.

## 2. Valorization of Olive Pomace 

Phenolic compounds derived from olive pomace are the bioactive compounds of interest in this study. Olive pomace comprises the main solid by-product of olive oil production, which is commonly utilized for olive pomace oil production, as combustible material, as animal feed, or it is directly disposed of into the environment without previous pretreatment [20]. Olive pomace is an interesting source of phenolic compounds, since only 1–2% of the total content of the phenolic compounds of olives goes into olive oil through its mechanical extraction production process (centrifugation of oil paste), while 53% and 45% of them remain in the liquid waste and the solid by-product (olive pomace), respectively [12]. Table 1 presents the total phenolic content and the main phenolic compounds of olive pomace.

**Figure 2:**
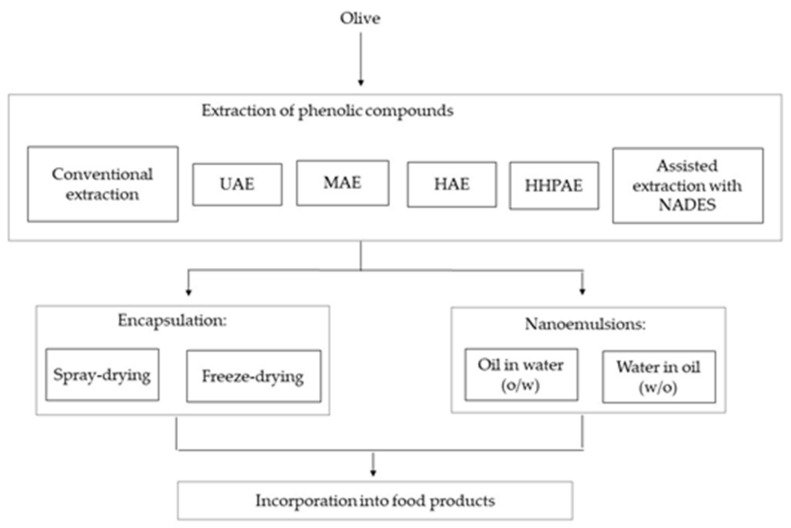
Flow chart for the integrated valorization approach of olive pomace based on the extraction of phenolic compounds and their protection by encapsulation. NADES: Natural Deep Eutectic Solvent

Figure 2 presents the proposed flow chart for an integrated valorization approach of olive pomace based on the extraction of phenolic compounds and their protection by encapsulation.

### 2.1. Extraction of Phenolic Compounds from Olive Pomace

Phenolic compounds have shown promising properties [14] associated with the protection of living systems from diseases, such as cardiovascular dysfunctions [14], as well as of food and pharmaceutical products against oxidation. The valorization of olive pomace is considered interesting for the recovery of its phenolic compounds. Environmentally-friendly and sustainable extraction techniques could be performed in order to ensure the high quality and antioxidant capacity of the phenolic compounds’ extracts. Therefore, innovative extraction methods, as previously presented, are proposed offering high extraction performance in a shorter time and requiring reduced energy. Table 2 summarizes representative studies about the extraction of phenolic compounds from olive pomace by using conventional and innovative techniques. These studies are comparatively discussed in the following sections with the experimental results of the current study.

#### 2.1.1. Conventional Extraction

Traditionally, conventional extraction techniques using organic solvents are applied. The execution of the solid-liquid extraction process is commonly performed in a Soxhlet apparatus, in which fresh solvent is repeatedly contacted with the solid matrix [38]. The main drawbacks of this technique are related to the need for large volumes of solvents, the long processing time, the absence of stirring, the requirement of a solvent evaporation stage, and the possible degradation of the vulnerable compounds due to the high extraction temperature used [39]. The extraction efficiency and the antioxidant potential are affected by the operation parameters, such as the extraction temperature, the extraction time, the liquid-to-solid ratio (L:S), and the type of solvent. The temperature should be optimized in order to enhance the mass transfer of phenolic compounds by increasing their diffusion rate into the solvent. The choice of optimum liquid-to-solid ratio also leads to promoted diffusion and increased extraction efficiency, since the high solvent volume enhances the extraction process [40].

The recovery of phenolic compounds from olive pomace has been performed by solid-liquid extraction methods by using various types of solvent including methanol, ethanol, acetone, water, and ethyl acetate [41]. Böhmer-Maas et al. [25] optimized the extraction of phenolic compounds from olive pomace using methanol as solvent at different concentrations of 40%, 60%, and 80% (*v*/*v*), different temperatures (45, 57.5, and 70 °C), and extraction times (60, 120, and 180 min). The TPC was promoted by using 40% (*v*/*v*) methanol at 70 °C and for 180 min, the antioxidant activity by using 40% (*v*/*v*) methanol at 45 °C and for 180 min, and the total determined individual phenols by HPLC by using 80% (*v*/*v*) methanol at 45 °C and for 180 min. Nakilcioğlu and Semih [24] studied the parameters of temperature (40, 50, and 60 °C), time (30, 60, and 90 min), and solvent type (methanol, ethanol, and acetone) for the extraction optimization of the phenolic compounds from olive pomace. They concluded that by applying an extraction temperature of 40 °C, extraction time of 89.49 min, and methanol as solvent type, the obtained extracts performed high TPC, antioxidant activity (DPPH), and concentration in individual phenolic compounds. It should be noted that the solubility of the target compounds increases with increasing temperature. Aludatt et al. [26] confirmed that the extraction of phenolic compounds from olive pomace at increased temperature (70 °C) achieved the maximum TPC and antioxidant activity. Čepo et al. [42] evaluated the effect of different extraction parameters including solvent types, extraction temperatures (20–90 °C), extraction times (30 min–24 h) and pH (2.0–10.3) of extraction solvent on TPC and antioxidant activity of extracts from olive pomace and they obtained high extraction yields and recovered extracts with strong antioxidant activity at optimum solvent extraction conditions (at 70 °C for 120 min by using 60% (*v*/*v*) ethanol as solvent).

In the current study, the effect of enzyme addition in citric buffer solution (pH 4.5) on the extraction of phenolic compounds from olive pomace has also been investigated. According to our results, the maximum TPC (23.06 mg GA/g olive pomace dw) and antioxidant activity (18.16 mg Trolox/g olive pomace dw) were achieved by using pectinase and polygalacturonase mixture 1% (*v*/*v*) in buffer as solvent at 60 °C for 4 h. Furthermore, by using these extraction parameters, the maximum concentration of oleuropein, hydroxytyrosol, rutin, and total determined phenolic compounds of the extracts were obtained, namely, 0.55, 0.93, 0.22, and 2.41 mg/g olive pomace dw, respectively [23].

#### 2.1.2. Ultrasound-Assisted Extraction (UAE)

UAE is strongly influenced by various processing parameters including time, temperature, liquid:solid ratio, power, and frequency [43]; therefore, their optimization is significant for the achievement of extracts with high quality in terms of antioxidant activity and individual phenolic compounds concentration. UAE achieves high TPC and strong antioxidant activity in a short extraction time suggesting the technique as a choice for the extraction of phenolic compounds from plant sources including wheatgrass [44] and black locust [45] as well as carotenoids from orange peels [46]. Regarding olive pomace, Goldsmith et al. [30] and Nunes et al. [31] proved that UAE was a very effective method for the extraction of phenolic compounds from olive pomace resulting in high yields in a short time. The UAE treatment was confirmed to be particularly effective for the recovery of hydroxytyrosol, maslinic acid, and oleanolic acid from olive pomace compared to the conventional one [32]. Tapia-Quirós et al. [33] effectively extracted phenolic compounds from olive pomace by applying UAE for 30 min with 50% (*v*/*v*) ethanol, proposing this technique as an ideal candidate for future increased evaluation. By combining ultrasounds with enzymes, the extraction efficiency of phenolic compounds from olive pomace can be further improved. For instance, Wang et al. [34] proved that the ultrasound-assisted enzymatic extraction resulted in higher extraction yields and extracts with stronger antioxidant activity than that obtained without the presence of enzymes. This could be attributed to the enzymatic degradation and rupture of the cell walls of the solid matrix, enhancing the performance of ultrasounds and increasing the yield of phenolic compounds in the final extracts.

#### 2.1.3. Microwave-Assisted Extraction (MAE)

The application of MAE for the recovery of phenolic compounds from olive pomace has been reported. Xie et al. [32] evaluated the application of MAE for the extraction of phenolic compounds, such as hydroxytyrosol, maslinic acid, and oleanolic acid from olive pomace. According to the results, the microwave treatment (600 W at 50 °C for 5 min) resulted in extracts with 8%, 24%, and 22% higher concentration of hydroxytyrosol, maslinic acid, and oleanolic acid, respectively, than those obtained by conventional extraction for 240 min. Tapia-Quirós et al. [33] and Jurmanović et al. [36] effectively extracted phenolic compounds from olive pomace by applying MAE at 90 °C for 5 min with 50% (*v*/*v*) ethanol, and for 10 min with 20% (*v*/*v*) ethanol, respectively.

According to our results, the combined microwave treatment and enzymes resulted in the high yield of phenolic compounds from olive pomace [23]. The microwave-assisted enzymatic extraction resulted in extracts from olive pomace with high TPC and stronger antioxidant activity. Moreover, the phenolic profiles of extracts revealed that the presence of enzymes enriched their phenolic content. Cellulase and pectinase hydrolyze the cell walls improving the release of target compounds; concluding that by applying the microwave-assisted enzymatic extraction at 60 °C for 30 min, extracts obtained were ~21% more enriched in phenolic compounds compared to those obtained by aqueous enzymatic extraction by the conventional method at 60 °C for 4 h (11.41 mg GA/g). 

#### 2.1.4. High Hydrostatic Pressure-Assisted Extraction (HHPAE)

HHPAE could be used as an alternative and sustainable tool for the recovery of phenolic compounds from olive pomace. Recently, Andreou et al. [47] reported that by applying 200 MPa for 10 min, the obtained extracts possessed high TPC (2.06 mg GA/L) and enriched phenolic compounds in terms of oleuropein, hydroxytyrosol, tyrosol, rutin, and luteolin concentrations. HHPAE promotes the penetration of solvent into cells allowing the release of phenolic compounds into the solvent.

#### 2.1.5. Extraction by Using NADES

A NADES consists of a mixture of a hydrogen bond acceptor (choline chloride) and a hydrogen bond donor, including carboxylic acids, amino acids, sugars, et cetera, formulating a eutectic mixture [48]. NADESs possess particular advantages in terms of physicochemical properties, such as adjustable surface tension and viscosity, as well as other characteristics including low toxicity, non-flammability, et cetera [49]. In particular, they have been suggested for the extraction of phenolic compounds as alternatives to the conventional organic solvents, offering both enhanced extraction efficiency and quality of the extracts [4,50].

According to our studies, the combination of innovative extraction techniques (UAE, MAE, HAE, and HHPAE) with NADES is proposed for the extraction of phenolic compounds from olive pomace. By applying UAE and a NADES composed of choline chloride and caffeic acid (CCA-mole ratio of 1:2), the TPC of extracts from olive pomace were 8% and 88%, higher than those obtained by 70% (*v*/*v*) ethanol and water, respectively. Moreover, the total determined phenolic compounds (oleuropein, hydroxytyrosol, caffeic acid, vanillin, rutin, and luteolin) of CCA extracts were significantly higher than those obtained by 70% (*v*/*v*) ethanol and water [4]. Similarly, de los Ángeles Fernández et al. [51] combined UAE and lactic acid, glucose, and 15% water as optimum NADES achieving high-efficiency in individual phenols such as hydroxytyrosol, tyrosol, apigenin, luteolin, et cetera in olive pomace. In the current study, it is also suggested that by combining microwaves with NADES, the extraction of phenolic compounds from olive pomace was favored. Microwave energy is effectively absorbed by NADESs, making these green solvents ideal for MAE treatments. The eutectic mixture of choline chloride and lactic acid (CLA-mole ratio of 1:2) achieved great extraction yields of phenolic compounds from olive pomace (29.57 GA/g dw) and the strongest antioxidant activity (17.51 g dw/g DPPH) compared to the ones obtained by 70% (*v*/*v*) ethanol and water [11]. High-speed shearing extraction based on the mass transfer due to the difference of the pressure among inside and outside cavities generated by the high speed of rotation is suggested to be a good alternative tool for the extraction of phenolic compounds from olive pomace by using NADES. According to our results, HAE and NADES influenced the quality of the obtained extracts. For instance, the eutectic mixture of choline chloride and caffeic acid (CCA-mole ratio of 1:2) at 60°C and 12,000 rpm possessed high TPC (34.08 GA/g dw) and strong antioxidant activity (5.11 g dw/g DPPH) compared to the ones obtained by 70% (*v*/*v*) ethanol and water. By applying these operating parameters and CCA, high concentrations of oleuropein, hydroxytyrosol, rutin, and the sum of the determined phenolic compounds were also achieved [11]. In our current study, by combining HHPAE and green solvent choline chloride and lactic acid (CLA-mole ratio of 1:2) at 600 MPa for 10 min, the extracts showed high TPC (25.96 mg GA/g dw) and antioxidant activity (15.67 g dw/g DPPH) [4]. Mitar and Kardum [28] and Garcia Borrego et al. [29] conventionally extracted phenolic compounds from olive pomace by using NADES and in particular, malic acid, D-fructose, and glycerol in 1:1:1 molar ratio and choline chloride-xylitol, and they confirmed higher extraction yields than those obtained by conventional solvents including 70% (*v*/*v*) ethanol and 80–50% (*v*/*v*) methanol, respectively.

### 2.2. Protection of Phenolic Compounds of Olive Pomace

#### 2.2.1. Microencapsulation (Freeze-Drying, Spray-Drying) of Phenolic Compounds from Olive Pomace

The microencapsulation technique is based on the formulation of dispersion or an emulsion containing the natural antioxidant (i.e., phenolic compounds) and an encapsulating agent followed by a drying process [23]. Various encapsulation agents, including polysaccharides (maltodextrins), have been used for the masking of phenolic compounds. Spray-drying and freeze-drying are the most commonly employed and studied encapsulation methods [52]. Through an integrated procedure, the microencapsulation of these vulnerable components is suggested in order to preserve their functionality and consequently facilitate their incorporation into functional food systems. Phenolic compounds from olive pomace possess low stability in environmental conditions, reduced bioavailability, limited water solubility, and rapid oxidation that restrict their incorporation into food formulations [53,54]. Therefore, they should be protected by using various microencapsulation techniques in order to overcome these disadvantages and to maintain their antioxidant activity [55].

The spray-drying technique is based on the atomization of a liquid formulation in hot air, producing a final product in powder form [53]. The final powdered products possess improved microbiological stability, limited degradation and oxidation mechanisms, and enhanced water solubility. Table 3 presents some studies based on the microencapsulation of phenolic compounds extracted from olive pomace. Paini et al. [54] encapsulated the phenolic compounds of olive pomace by applying the spray-drying technique and using maltodextrin as an encapsulating agent. They concluded that different encapsulation parameters, such as inlet temperature, the concentration of maltodextrin, et cetera, affected the properties of the final products. By increasing the concentration of maltodextrin, the powder appeared to lower bulk density and higher microparticle size. The final powder possessed enhanced stability at storage conditions and significant antioxidant activity. Moreover, Aliakbarian, Paini, and Albertom [56] investigated the effect of different ratios of maltodextrin (MD) and gum Arabic (GA) as encapsulating coating (0:100, 20:80, 40:60, 60:40, 80:20, and 100:0% *w*/*w*) on the encapsulation efficiency and the physical and antioxidant properties of the final products derived from phenolic compounds of olive pomace. The MD:GA ratio of 60:40 led to the formulation of powders with improved water solubility and minimal losses of phenolic content during the drying process. Cepo et al. [27] encapsulated phenolic compounds from olive pomace by using cyclodextrin as agent and spray-drying method. The final products possessed increased TPC and remarkable antioxidant protection in oil and meat models (0.1–3%) that was similar to those obtained by synthetic antioxidant BHA. Similarly, Jurmanović et al. [36] encapsulated phenolic compounds from olive pomace by using the spray-drying technique (inlet temperature: 130 °C and aspirator rate: 100%) ensuring satisfactory yields and powder characteristics.

The microencapsulation of phenolic compounds from olive pomace has also been effectively carried out by applying the freeze-drying technique. According to our results, by freeze-drying the phenolic compounds extracts with maltodextrin as a coating agent final powdered products were developed with increased stability, improved properties in terms of moisture, hygroscopicity, water solubility, and antioxidant activity, and high microencapsulation efficiency, promoting their incorporation into food systems [23].

#### 2.2.2. Protection of Phenolic Compounds of Olive Pomace by Incorporation in Nanoemulsion Formulations

The main challenge in developing functional foods is associated with the incorporation of bioactive compounds in food matrices, using edible delivery systems, which are capable of encapsulating, protecting, and releasing the bioactive compounds and are also suitable for the food industry. In the case of lipophilic bioactive compounds, their incorporation into food products is limited due to some major factors, such as poor water solubility, chemical instability, poor bioavailability, and high melting point (crystalline at ambient temperature) [57]. Nanoemulsions as carriers for phenolic compounds have gained interest due to their unique functional characteristics and physicochemical properties, such as high physical stability and optical clarity/transparent appearance, as well as enhanced bioavailability [58,59,60,61,62]. Nanostructured systems also offer a great number of advantages due to their nanostructure. The nanosize of the droplets provides high encapsulation efficiency and stability, prevention against chemical reactions, enhanced solubility, distinct control release, as well as regulation of digestion rate and uptake in the gastrointestinal tract due to their area-to-volume ratio; their physicochemical behavior is significantly different from those at micro- and macroscales. By reducing the oil droplet size into the nanometric scale, the solubility of the bioactive is significantly increased.

Nanoemulsion delivery systems can easily incorporate them in aqueous-based foods, such as many beverages, dressings, desserts, dips, sauces, and yogurts [63]. However, it is important that while the nanoemulsion delivery systems protect the encapsulated bioactive compounds, they should also maintain their appearance and physicochemical properties (droplet size and charge, transparency, and viscosity) during their shelf-life. Therefore, in order to assess the adequacy of an encapsulation system, it is critical to monitor not only its encapsulation stability, but also its mean droplet diameter growth during storage. In particular, various researchers proved that emulsion delivery systems can protect the phenolic compounds presenting high encapsulation stability values during one month of storage, as can be seen in Table 4. Nanoemulsion formulations have been developed using different emulsification techniques (high pressure, ultrasonic homogenizer) and numerous lipid phases (vegetable, fish, and essential oil) as they play a crucial role during homogenization. Depending on the emulsification conditions, the limited incorporation of oxygen during emulsification, and also the emulsifier concentration and dispersed phase volume used, the encapsulation stability of phenolic compounds can range up to 73% during storage at 25 °C [16,64,65,66]. During the extensive emulsification process (energy, time) there could be an increase in local temperature and a large amount of air incorporated into the system, which promotes oxidative degradation of polyphenols [60]. Compared to different types of fats and oils examined, vegetable oils, such as extra virgin olive oil and olive pomace oil have received the most attention in nanoemulsion formulation because they are widely known for their beneficial properties, their high nutritional values, as well as for their stability during heat treatment or storage. Moreover, these vegetable oils were proved to be an excellent choice as a lipid phase for emulsions and double emulsions [64,67,68,69], producing nanoemulsions with high physical and chemical stability during storage [69,70].

Comparing the two types of nanoemulsions (oil-in-water (o/w), water-in-oil (w/o)), the o/w nanoemulsions were usually more transparent with satisfactory physicochemical properties; low droplet size diameter and narrow droplet distribution combined with high absolute ζ-potential values, presenting a good indication for the high kinetic stability of the system. As far as the physicochemical properties of w/o nanoemulsions, their droplet size is often higher and near to 500 nm with turbid appearance. Moreover, water-in-oil emulsions present a larger extent of phase separation in comparison to o/w nanoemulsions, and also higher droplet growth. Comparing the homogenization mechanisms (Table 4), ultrasonication and high-pressure homogenization are the most efficient and suitable emulsification techniques for the production of nanoemulsions [61].

As far as their encapsulation stability is concerned, both emulsion types exhibited good phenolic compounds retention. In particular, the proper combination of emulsion composition and homogenization condition can result in nanoemulsions with the highest encapsulation stability for 30 days of storage. For o/w nanoemulsions, various researchers claimed that as the dispersed phase increases, and consequently the droplet size increases, the chemical stability increases because the aqueous phase and water-soluble prooxidants decrease [77,78]. Regarding the w/o nanoemulsions, their chemical stability is high during storage due to the limited concentration of the aqueous phase and the presence of minor compounds of the lipid phase with antioxidant activity. Furthermore, the type of emulsifier may affect the repulsive or attractive interactions between antioxidants and droplets, and these interactions are responsible for the localization of antioxidants in the emulsion interface [17]. Finally, the number and location of phenolic hydroxyl groups and benzene methyl groups affect the antioxidant activity of phenolic compounds. Some of them have more surface activity or they can bind to surface-active emulsifiers, so they can be located at the oil-water interface where lipid oxidation usually occurs. The location of the phenolic compounds in an emulsion plays an important role in determining its physical and oxidative stability [65]. Thus, the nanoemulsions enriched with polyphenols generally present good chemical and encapsulation stability combined with the limited appearance of creaming or sedimentation during storage could be due to the synergistic action of polyphenolic compounds and non-ionic emulsifiers which enhance droplets’ steric repulsion [76,77]. Moreover, according to Maqsoudlou et al. [80], the incorporated phenolic compounds in nanoemulsions present enhanced bioavailability in comparison with non-encapsulated ones.

## 3. Conclusions

The valorization of olive pomace is a promising approach in order to deal with the economic and environmental issues enhancing the profitability of the olive oil sector. The recovery of phenolic compounds could be performed by novel extraction processes improving the quality of the extracts in terms of antioxidant potential and phenolic content. The combination of natural deep eutectic solvents (NADESs) with innovative extraction-assisted methods using ultrasound (UAE) microwave (MAE), homogenization (HAE), and high hydrostatic pressure (HHPAE) proved to be effective for the extraction of phenolic compounds from olive pomace. NADESs studied, especially choline chloride-caffeic acid (CCA) and choline chloride-lactic acid (CLA), enhanced the extraction of phenolic compounds as compared with conventional solvents. CCA showed high total phenol content (TPC) by applying HAE at 60 °C/12,000 rpm and by UAE at 60 °C. CLA was the best NADES for the MAE at 60 °C and HHPAE at 600 MPa/10 min. The results of the current study suggest the phenolic compounds recovery from olive pomace by applying NADESs and innovative extraction techniques on an industrial scale.

Currently, both freeze-drying and nanoemulsion-based encapsulation present satisfactory results for protecting natural antioxidants. Generally, the use of the nanoemulsion technique for encapsulation of natural antioxidants is considered a more promising approach due to its advantage over other encapsulation techniques, namely, transparent appearance, controlled release, adjustable rheology, and high physical stability. As phenolic compounds present surface activity, they modified the physical properties of the o/w and w/o nanoemulsions, such as reduction of the mean droplet diameter during homogenization and therefore increased stability. The emulsifier’s concentration and type affected the localization and the stability of incorporated phenolic compounds. The w/o nanoemulsions presented great encapsulation stability during storage exhibiting low kinetic stability. The o/w nanoemulsions were physically stable during storage without extensive phase separation, however, the degradation of the polyphenolic compounds was higher. 

However, further research and improvements will be required in the future in order to overcome their limitations.

## Figures and Tables

**Table 1:** Total phenolic content and the main phenolic compounds of olive pomace.

Total Phenolic Content (mg of gallic acid (GA)/g dw)		Reference
10.2 to 40.0		[21]
Main phenolic compounds (mg/g dw)		
Hydroxytyrosol	0.61–8.70	[4,22]
Oleuropein	1.22–13.50	
Vanillin	0.92–3.64	
Apigenin	0.41–0.60	
Rutin	0.21–1.70	
Luteolin	0.02–0.14	

**Table 2:** Extraction of phenolic compounds from olive pomace by conventional and innovative techniques.

Optimum Extraction Parameters	Extraction Efficiency of Extracts		Reference
Conventional extraction			
Solvent: citric buffer pH = 4.5 and 1% enzyme solution in volume (*v*/*v*) Temperature: 40 °C Extraction time: 4 h L:S: 12.5:1 mL/g Apparatus: Water bath	TPC: 11.41 mg GA/g dw	DPPH: 24.17 mg Trolox/g dw	[23]
	oleuropein: 0.55 mg/g dw; hydroxytyrosol: 0.93 mg/g dw; rutin: 0.22 mg/g dw; total determined phenolic compounds by HPLC: 2.41 mg/g dw		
Solvent: Methanol Temperature: 40 °C Extraction time: 89.49 min L:S: 2:1 mL/g Apparatus: Water bath	TPC: 210 mg GA/kg dw	DPPH: 16.97%	[24]
	hydroxytyrosol: 24.29 mg/kg dw; syringic acid: 0.68 mg/kg dw; oleuropein: 33.22 mg/kg dw		
Solvent: 40% and 80% (*v*/*v*) methanol Temperature: 45 and 70 °C Extraction time: 180 min Apparatus: Water bath	TPC: 23.06 mg GA/g dw	DPPH: 20.41 mg Trolox/g dw	[25]
	hydroxytyrosol: 154.90 mg/kg dw; tyrosol: 1115.40 mg/kg dw; syringic acid: 153.20 mg/kg dw; total determined phenolic compounds by HPLC: 1481.30 mg/kg dw		
Solvent: Methanol Temperature: 60 °C Extraction time: 12 h Apparatus: Water bath	TPC: 4.07 mg GA/g dw	DPPH: 76.67%	[26]
	protocatechuic acid: 16.3%; syringic acid: 3.10%; vanillic acid: 4.60%; rutin: 24.60%; hesperidin: 23.50%		
Solvent: 60% (*v*/*v*) ethanol Temperature: 70 °C Extraction time: 120 min L:S: 5:1 mL/g Apparatus: Water bath	TPC: 3.62 mg GA/g	DPPH: 3.64 mg Trolox/g	[27]
	hydroxytyrosol: 81.80 mg/kg; tyrosol: 86.05 mg/kg; oleuropein: 115.14 mg/kg		
Solvent: Malic acid (Ma), D-fructose (Fru), and Glycerol (Gly) Temperature: 60 °C Extraction time: 2 h Apparatus: Magnetic stirrer	TPC: 15.02 mg GA/g dw		[28]
Solvent: choline chloride-xylitol Temperature: 40 °C Extraction time: 1 h L:S: 1:1 mL/g Apparatus: Magnetic stirrer	TPC: ~20.00 mg GA/g dw		[29]
**Ultrasound-assisted extraction**			
Solvent: Water Temperature: 30 °C Power: 250 W Frequency: 50 Hz Extraction time: 75 min L:S: 50:1 mL/g Apparatus: ultrasonic bath	TPC: 19.71 mg GA/g	DPPH: 31.23 mg Trolox/g	[30]
	total determined phenolic compounds by HPLC: 62.05 μg tyrosol/g		
Solvent: Water Temperature: 25 °C Power: 160 W Frequency: 20 KHz Extraction time: 5 min L:S: 50:1 mL/g Apparatus: Multi-frequency Multimode Modulated (MMM) ultrasonic device	TPC: 402 µg GA/mL	DPPH≈ 1.180 µg TE/mL	[31]
	hydroxytyrosol: 83.60 mg/100 g; tyrosol: 3.40 mg/100 g		
Solvent: 90% (*v*/*v*) ethanol Temperature: 50 °C Frequency: 20 kHz Extraction time: 3 min L:S: 30:1 mL/g Apparatus: ultrasonic probe	hydroxytyrosol: 55.11 mg/g; maslinic acid: 381.20 mg/g; oleonolic acid: 29.80 mg/g		[32]
Solvent: 50% (*v*/*v*) ethanol Temperature: 20 °C Extraction time: 30 min L:S: 20:1 mL/g Apparatus: ultrasonic bath	TPC: 8.05 mg GA/g	ABTS: 31.63 mg Trolox/g	[33]
Solvent: disodium hydrogen phosphate-citric acid buffer Enzymes: cellulase, hemicellulase and pectinase Temperature: 55 °C Power: 200 W Frequency: 40 kHz Extraction time: 40 min pH: 5.75 L:S: 4:1 mL/g Apparatus: ultrasonic bath	Phenolic compounds yield: 4%		[34]
Solvent: Choline chloride-caffeic acid (CCA) Temperature: 60 °C Power: 280 W Frequency: 60 kHz Extraction time: 30 min L:S: 12.5:1 mL/g Apparatus: ultrasonic bath	TPC: 20.14 mg GA/g dw	DPPH: 20.69 g dw/g DPPH	[4]
	oleuropein: 0.85 mg/g dw; hydroxytyrosol: 1.05 mg/g dw; rutin: 0.40 mg/g dw; total determined phenolic compounds: 2.51 mg/g dw		
Solvent: Lactic acid, glucose and 15% water Temperature: 40 °C Power: 200 W Frequency: 20 kHz Extraction time: 30 min L:S: 75:1 mL/g Apparatus: ultrasonic bath	apigenin: 0.08 mg/g dw; hydroxytyrosol: 0.11 mg/g dw; rutin: 0.01 mg/g dw; luteolin: 0.45 mg/g dw		[35]
**Microwave-assisted extraction**			
Solvent: 90% (*v*/*v*) ethanol Temperature: 50 °C Power: 600 W Frequency: 2.45 GHz Extraction time: 5 min L:S: 30:1 mL/g	hydroxytyrosol: 53.20 mg/g; maslinic acid: 356.00 mg/g; oleonolic acid: 26.30 mg/g		[32]
Solvent: 50% (*v*/*v*) ethanol Temperature: 90 °C Extraction time: 5 min L:S: 20:1 mL/g	TPC: ~10.00 mg GA/g		[33]
Solvent: 20% (*v*/*v*) ethanol Power: 700 W Extraction time: 10 min L:S: 50:1 mL/g Apparatus: closed-vessel microwave extraction system	TPC: 50.18 mg GA/g dw	DPPH: 45.42 mg Trolox/g dw	[36]
	oleuropein: 0.03 mg/g dw; hydroxytyrosol: 1.22 mg/g dw; tyrosol: 0.13 mg/g dw		
Solvent: Citric acid buffer Enzyme: pectin lyase and polygalacturonase Temperature: 60 °C pH: 4.5 Power: 400 W Extraction time: 30 min L:S: 12.5:1 mL/g Apparatus: laboratory microwave equipment	TPC: 14.37 mg GA/g dw	DPPH: 20.23 g dw/g DPPH	[23]
	oleuropein: 0.55 mg/g dw; hydroxytyrosol: 1.02 mg/g dw; rutin: 0.23 mg/g dw; total determined phenolic compounds by HPLC: 2.46 mg/g dw		
Solvent: Choline chloride-lactic acid (CLA) Temperature: 60 °C Power:280 W Frequency: 60 kHz Extraction time: 30 min L:S: 12.5:1 mL/g Apparatus: laboratory microwave equipment	TPC: 29.57 mg GA/g dw	DPPH: 17.51 g dw/g DPPH	[4]
	oleuropein: 7.56 mg/g dw; hydroxytyrosol: 0.89 mg/g dw; rutin: 0.74 mg/g dw; total determined phenolic compounds by HPLC: 9.49 mg/g dw		
**Homogenate-assisted extraction**			
Solvent: Choline chloride-caffeic acid (CCA) Temperature: 60 °C Homogenization speed: 12,000 rpm Extraction time: 30 min L:S: 12.5:1 mL/g Apparatus: high speed homogenizer	TPC: 34.08 mg GA/g dw	DPPH: 5.11 g dw/g DPPH	[4]
	oleuropein: 12.86 mg/g dw; hydroxytyrosol: 3.37 mg/g dw; rutin: 1.71 mg/g dw; total determined phenolic compounds by HPLC: 18.30 mg/g dw		
**High hydrostatic pressure-assisted extraction**			
Solvent: 50% (*v*/*v*) ethanol Pressure: 200 MPa Extraction time: 10 min L:S: 10:1 mL/g Apparatus: high pressure unit	TPC: 2.06 mg GA/L		[37]
	oleuropein: 84.65 mg/L; hydroxytyrosol: 2001.56 mg/L; tyrosol: 124.88 mg/L; rutin: 17.59 mg/L; luteolin: 39.27 mg /L		
Solvent: Choline chloride-lactic acid (CLA) Pressure: 600 MPa Extraction time: 10 min L:S: 12.5:1 mL/g Apparatus: high pressure unit	TPC: 25.96 mg GA/g dw	DPPH: 15.67 g dw/g DPPH	[4]
	oleuropein: 1.94 mg/g dw; hydroxytyrosol: 2.57 mg/g dw; rutin: 0.66 mg/g dw; total determined phenolic compounds by HPLC: 5.31 mg/g dw		

L:S: liquid: solid ratio; TPC: total phenolic content; DPPH: antioxidant radical scavenging by DPPH (2,2-diphenyl-1-picryl-hydrazyl-hydrate) assay; ABTS: antioxidant radical scavenging by ABTS (2-azino-bis(3-ethylbenzothiazoline-6-sulfonic acid) assay; HPLC: high-performance liquid chromatography

**Table 3:** Microencapsulation of phenolic compounds from olive pomace.

Microencapsulation Conditions	Microencapsulation Performance		References
Technique: Spray-drying Agent: Maltodextrins (MD) 16.5–19.5 DE MD concentration: 100 g/L Air flow: 30 m^3^/h Inlet Temperature: 130 °C Feed Flow: 10 mL/min	TPC: 39.5 mg CA/g dw	DPPH: 33.8 mmol DPPH/L extract	[54]
	Encapsulation yield: 87.3%; Microencapsulation efficiency: 76%; Water solubility: 85%		
Technique: Spray-drying Agent: Hydroxypropyl-*β*-cyclodextrin Inlet Temperature: 130 °C Aspirator: 100% Feed Flow: 6.5 mL/min	TPC: 13.57 mg GA/g dw	DPPH: 17.85 mg Trolox/g dw	[36]
	Encapsulation yield: 82.40%; Mean spherical diameter: 3.66 μm		
Technique: Spray-drying Agent: Maltodextrins (MD) 16.5–19.5 DE and gum arabic (GA) MD:GA ratio: 60:40 MD concentration: 100 g/L Air flow: 30 m^3^/h Inlet Temperature: 160 °C Feed Flow: 5 mL/min	TPC: 36.9 mg CA/g dw	DPPH: 12.5 mmol DPPH/L extract	[56]
	Encapsulation yield: 94%; Water solubility: 69.4%		
Technique: Freeze-drying Agent: Maltodextrin (MD) 19DE Phenolic compounds: MD Ratio: 1:20 *w*/*w*	DPPH: 0.69–1.25 mg Trolox/g dw		[23]
	Encapsulation efficiency: 82–90%; Water solubility: 91–97%, Hygroscopicity: 7–23 g H_2_O/100 g dw		
Technique: Spray-drying Agent: *β*CD, HP*β*CD, RAMEB, or *γ*CD Air flow: 500 L/h Inlet Temperature: 120 °C Air pressure: 6 bar Feed Flow: 5 mL/min	Antioxidant protection: 0.1–3%; Antioxidant activity: HP*β*CD: 1.242 mg/g of Trolox equivalents and RAMEB: 1.422 mg/g of Trolox equivalents		[27]

DE: dextrose equivalent

**Table 4:** Emulsion and nanoemulsion delivery systems for polyphenols reported in the literature.

Emulsification Techniques	Emulsifier and Lipid Phase	Droplet Size t_0_	Droplet Size t_storage_	Encapsulation Stability/Storage Conditions	References
**Oil-in-water nanoemulsion (o/w)**					
High shear homogenization High-pressure homogenizer	Whey protein isolate (WPI) Sunflower oil	220 nm	Relatively constant	50% phenolic content remaining after 26 days at 25 °C	[71]
High shear homogenization	Tween 20 Refined olive oil	-	Limited phase separation	80% gallic acid remaining after 10 days at 25 °C	[72]
High shear homogenization Microfluidization	Whey protein isolate (WPI) Flaxseed oil	220–224 nm	Relatively constant	73% phenolic extracts remaining after 27 days at 25 °C 46% phenolic content remaining after 35 days at 25 °C	[73]
High shear homogenization High-pressure homogenizer	Caseinate, Tween 20 Kenaf seed oil	130 nm	133.85 nm	46% polyphenolic content remaining after 56 days at 25 °C	[74]
High shear homogenization High-pressure homogenizer	Tween 20 Soybean oil	1.29–1.43 μm	1.48–1.78 μm	91–98% gallic acid remaining after 7 days at 25 °C	[61]
High shear homogenization High-pressure homogenizer	Lipophilic soy lecithin, sugar ester, Tween 20 Peanut oil	128.2–211 nm	Relatively constant	encapsulated antioxidants were stable after 20 days at 25 °C	[75]
**Water-in-oil nanoemulsion (w/o)**					
High shear homogenization High-pressure homogenizer	Tween 20 Soybean oil	2.99–5.36 μm	4.1–6.99 μm	99–98% gallic acid remaining after 7 days at 25 °C	[61]
High shear homogenization	Tween 20 Refined olive oil or extra virgin olive oil	150–800 nm	Relatively constant	50–25% polyphenolic content remaining after 20 days at 25 °C	[76]
High shear homogenization	Tween 20, Span 80 Extra virgin olive oil or Sunflower oil	0.68–0.93 µm	~0.9–1 µm	92–97% polyphenolic content remaining after 30 days at 25 °C	[77]
High shear homogenization Ultrasonication	Span 80 Mustard oil	29–621 nm	-	80.63–88.76% polyphenolic content remaining after 30 days at 25 °C	[78]
High shear homogenization	Span 80 Soybean oil	1 μm	-	encapsulated antioxidants were relatively stable after 20 days at 25 °C	[79]

## Data Availability

Not Applicable.
